# *De novo* Nanopore Genome Sequencing of the Clinical *Diutina catenulata* Type-strain CBS565

**DOI:** 10.1007/s11046-022-00632-x

**Published:** 2022-05-10

**Authors:** Sander Boden, Florent Morio, Miaomiao Zhou, Bert Gerrits van den Ende, Ferry Hagen

**Affiliations:** 1 Westerdijk Fungal Biodiversity Institute, Uppsalalaan 8, 3584 CT Utrecht, The Netherlands; 2 Avans University of Applied Sciences, Breda, The Netherlands; 3 Nantes Université, CHU de Nantes, Cibles et Médicaments des Infections et de l’immunité, IICiMed, UR1155, 44000 Nantes, France; 4 Department of Medical Microbiology, University Medical Center Utrecht, Heidelberglaan 100, 3584 CX Utrecht, The Netherlands

**Keywords:** *Diutina catenulata*, Antifungal resistant, Emerging pathogen, Long-read sequencing, Nanopore sequencing

## Abstract

*Diutina catenulata* is an ascomycetous yeast, that is regularly fluconazole-resistant and increasingly reported as the cause of invasive infection in humans. Here, we describe the de novo genome assembly of the clinical *D. catenulata* type-strain CBS565 and provide insights into the genome and compared it to an Illumina-sequenced environmental strain.

**Supplementary Information:**

The online version contains supplementary material available at 10.1007/s11046-022-00632-x.

*Diutina catenulata* is an ascomycetous yeast belonging to the *Saccharomycetales* and a member of the *Debaryomycetaceae*/*Metschnikowiaceae* clade [1, 2]. It was originally described in 1942 as *Candida catenulata* but recently assigned to the novel genus *Diutina* that presently accommodates six other species *D. neorugosa*, *D. pseudorugosa*, *D. ranongensis*, *D. rugosa*, *D. scorzettiae* and *D. siamensis* [1]. *Diutina* yeasts have been recognized as emerging pathogens [3, 4]. Molecular characterization and antifungal susceptibility testing of the clinically relevant *Diutina* species showed that *D. mesorugosa* was in fact indistinguishable from *D. rugosa*, thus the former was found to be a synonym for the latter [3]. *Diutina catenulata* has been mainly reported from birds and contaminated cheeses but this species, which frequently display high fluconazole MIC-values is also increasingly reported as a cause of invasive infection in humans [4–6]. Here, we provide the de novo genome assembly and annotation of the clinical *D. catenulata* type-strain CBS565.

The *D. catenulata* type-strain CBS565 was isolated in 1926 from faeces of a patient with dysentery who at that time lived in Puerto Rico [1]. Genomic DNA extraction was performed as previously described [7]. The nanopore sequencing library preparation was performed with the ligation sequencing kit (SQK-LSK109; ONT, Oxford, UK) and the native barcoding kit (EXP-NBD114; ONT). The two libraries were run onto a MinION flow cell (FLO-MIN106; ONT) following the manufacturer’s protocol.

The raw nanopore reads were basecalled using Guppy v5.0.16 + b9fcd7b5b (ONT) using the settings—flowcell FLO-MIN106—kit SQK-LSK109—barcode_kits EXP-NBD114—device cuda:0, followed by demultiplexing and barcode trimming with the same software. De novo genome assembly was performed with Flye v2.9 (https://github.com/fenderglass/Flye; [8]) using the parameters—nano-raw < fastq > —out-dir < directory > —genome-size 13 m.

The assembled genome quality was assessed using GenomeQC [9]. The total genome size was 14,464,696 bp with an N50 of 2,438,920 bp, distributed over 9 contigs (range 3,918,888–370,337 bp; coverage of 107–138X) and a circular mitochondrial genome of 20,926 bp (3,528X coverage). Percentages of adenine, thymine, cytosine and guanine were 23.36, 23.37, 26.69 and 26.58%, respectively, resulting in a GC-content of 53.27%, which is close to 54.5% as previously determined by DNA–DNA-reassociation analyses [1, 10].

Annotation was performed using the Funannotate pipeline v1.8.9 (https://github.com/nextgenusfs/funannotate). Ab initio gene prediction was done using GeneMark-ES v4 [11], Augustus v3.3.3 [12], SNAP [13] and GlimmerHMM v3.0.4 [14]. The latter three were trained with validated protein predictions found by BUSCO v2.0 [15]. Functional annotation was performed using InterProScan v5.52-86.0 [16] and SignalP v5.0 [17] as part of the Funannotate pipeline. The final annotated genome contained 5,209 predicted genes, consisting of 4918 mRNAs and 291 tRNAs. Among the 106 CAZymes predicted, 65 were observed by SignalP to be extracellular, for the 177 predicted proteases 65 were found to be extracellular.

We used Mauve [18] to compare the de novo assembly of CBS565 to that of the published genome of the environmental strain WY3-10-4 [19], which showed various structural variations (Fig. 1). The Average Nucleotide Identity (ANI) was calculated using Oat [20], this yielded an ANI of 98.06%.

**Fig. 1:**
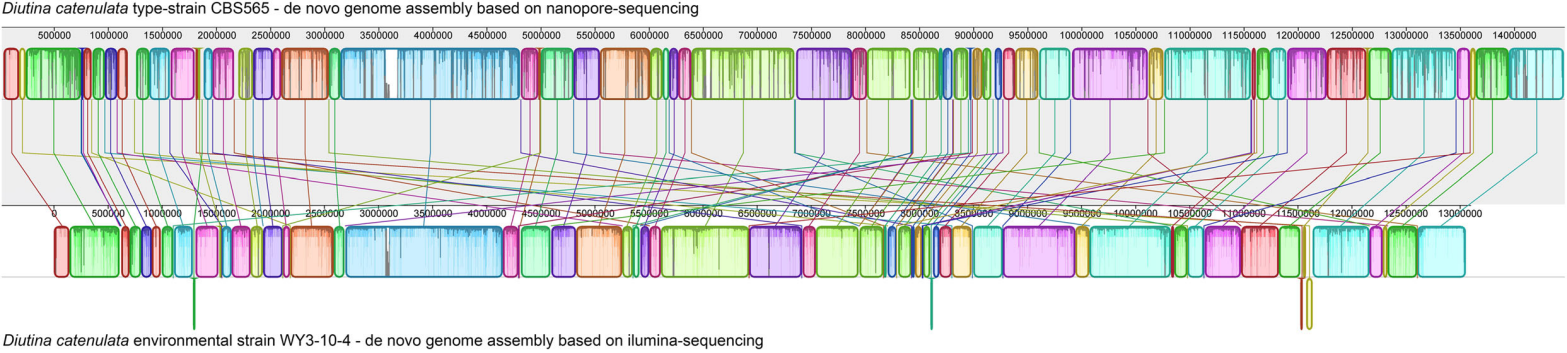
Genome alignment of *Diutina catenulata* strains CBS565 and WY3-10-4. Mauve [18] was used with default LCB weight settings to visualize the de novo genome assembly of the nanopore-sequenced strain CBS565 (upper lane) versus that of the recently published de novo assembled illumina-sequenced strain WY3-10-4 [19] (lower lane). Homologues sequence-segments (coloured boxes) are connected by a line, and box that is downward indicates an inverted segment compared to the other genome Numbers indicate sequence-length in base pairs

With Orthofinder [21] the CBS565 and WY3-10-4 proteomes were clustered using the default parameters. In total 4577 orthologous clusters were built. A total of 129 unique putative proteins in CBS565 and 550 in WY3-10-4 were found. Some well-known virulence factors of *Candida albicans* (e.g., proteases, hyphal wall protein, agglutinin-like sequence protein) were used to identify virulence factors in CBS565 by BLAST [22]. At least one copy of these virulence factors were found in the genome of CBS565 (Supplementary Data).

## Supplementary Information

Below is the link to the electronic supplementary material.

Supplementary file1 (XLSX 1449 kb)

Supplementary file2 (XLSX 9 kb)

## Data Availability

The data has been deposited in NCBI Genomes. BioProject accession number PRJNA770350, BioSample accession number SAMN22215821, and sequencing reads with SRA accession numbers SRR16292071 and SRR16311897.
